# Mycoviruses of an endophytic fungus can replicate in plant cells: evolutionary implications

**DOI:** 10.1038/s41598-017-02017-3

**Published:** 2017-05-15

**Authors:** L. Nerva, G. C. Varese, B. W. Falk, M. Turina

**Affiliations:** 1 Institute for Sustainable Plant Protection, CNR, Strada delle Cacce 73, 10135 Torino, Italy; 2 Mycotheca Universitatis Taurinensis (MUT), Department of Life Sciences and Systems Biology, University of Turin, Viale Mattioli 25, 10125 Torino, Italy; 3 Plant Pathology Department, University of California Davis, Davis, CA 95616 USA

## Abstract

So far there is no record of a specific virus able to infect both fungal and plant hosts in nature. However, experimental evidence shows that some plant virus RdRPs are able to perform replication *in trans* of genomic or DI RNAs in the yeast *Saccharomyces cerevisiae*. Furthermore, tobacco mosaic virus was recently shown to replicate in a filamentous ascomycetous fungus. Thus, at least experimentally, some plant viruses can infect some fungi. Endophytic fungi have been reported from many plants and several of these fungi have been shown to contain viruses. Here we tested if mycoviruses derived from a marine plant endophyte can replicate in plant cells. For this purpose, we used partially purified viral particles from isolate MUT4330 of *Penicillium aurantiogriseum* var. *viridicatum* which harbors six virus species, some having dsRNA and some positive-strand ssRNA genomes. These were transfected into three distinct plant protoplast cell systems. Time-course analysis of absolute RNA accumulation provided for the first time evidence that viruses of two species belonging to the *Partitiviridae* and *Totiviridae* families, can replicate in plant cells without evidence of host adaptation, i.e, changes in their nucleotide sequence.

## INTRODUCTION

Interest in viruses is mostly based on their ability to cause disease in plants, animals and humans, but in recent decades their role in mutualistic symbiosis has been shown^1^. Viruses comprise a heterogeneous group of microorganisms; their diversity suggests multiple origins of viruses and thus they have no conserved sequence that can be used for general detection of undescribed species. Nevertheless, metagenomics approaches and new bioinformatics tools have increased exponentially our ability to detect and describe new viruses^2–4^. Thanks to these approaches new viral species from metagenomics samples were described helping to enrich the virus taxonomic framework. An example of how metagenomics is rapidly changing our perception of the viromes associated with higher organisms was recently shown for the invertebrate virosphere^4^.

This enriched taxonomic framework shows a number of clades (often at the family or genus taxonomic level) which have as hosts a mosaic of species belonging to different kingdoms^4^. Further interesting data come from metagenomics studies of plant samples in natural environments. The most abundant species identified belong to the families *Totiviridae*, *Partitiviridae*, *Endornaviridae* and *Chrysoviridae* ^5^. Recently, also a circular single stranded DNA virus in the *Genomovirus* genus was shown to replicate both in insect and fungal cells^6^. All the above mentioned families have members which are reported as persistent plant viruses lacking the typical movement protein but able to replicate in meristematic cells leading to their ability to infect all plant tissues; specific members of these taxonomic clades can also infect fungi^7^ but none of the distinct viral species in these families have been shown to be able to infect both plants and fungi. It is important to note that several of these viruses are from endophytic fungi, thus they reside in fungal hosts which reside in plants. A link between plant viruses and mycoviruses was for the first time shown for viruses of the *Hypoviride* family: analysis of protein sequences revealed that hypoviruses share a common ancestor with the plant-infecting Potyviruses^8^. In addition, a still unrecognized group of viruses formed by Ourmia-like fungal viruses^9^, plant ourmiaviruses^10^, and a number of new species of still undefined host constitute a well-supported clade in phylogenetic analysis^4, 11^. Two other families that show a wide mosaic of hosts are *Bunyaviridae* ^12^ and *Reoviridae* ^13^. In these two families we can find viruses infecting animals (vertebrate and invertebrate), plants and fungi. This evidence suggests the possibility that common host cellular machinery for virus replication is shared between these different hosts.

Further evidence of relationships between fungal and plant viruses comes from the family *Narnaviridae*. This family of mycoviruses is divided into two genera based on subcellular localization: *Narnavirus*, which has members that are cytosolic viruses infecting yeasts and oomycetes, and *Mitovirus* which has members which are mitochondria-confined viruses found only in fungi^14^; however, some mitovirus sequences have been found to be endogenized into plant genomes with almost complete sequences suggesting one or more integration events of fungal mitoviruses into the chromosomal DNA of vascular plants^15^.

Experimental evidence of the ability of viruses to adapt their replication machinery to host cells of species belonging to different kingdoms are already present in literature. The first example was shown by the ability of brome mosaic virus (BMV) to replicate in the yeast, *Saccharomyces cerevisiae* ^16^. Another example comes from work showing the ability of two replicase proteins, p33 and p92, of tomato bushy stunt virus (TBSV) to maintain and replicate virus-associated defective interfering (DI) RNAs^17^. This evidence has confirmed that at least these plant viral RdRPs can function in yeast cells. Another recent work reports evidence of tobacco mosaic virus (TMV) replication in three species of fungi in the genus *Colletotrichum* ^18^. Overall, these data seem to confirm that some plant viruses can replicate in cells of different ascomycetous species. These experiments were stimulated by the aim to use a genetically treatable host such as yeast for plant viruses^19^.

Despite these reports of plant viruses replicating in fungal cells, surprisingly, to date there are still no reports on replication of mycoviruses in plant cells. This is surprising particularly in light of the fact that viruses infecting fungal endophytes must also be continuously exposed to the plant cells which are hosts for the fungus. For this reason we decided to test the ability of a number of representative mycoviruses to infect plant cells. In previous work^2^ we reported 12 new mycovirus species isolated from a collection of marine fungi associated with the marine plant *Posidonia oceanica* ^20, 21^. One isolate of this fungal collection, *Penicillium aurantiogriseum* var. *viridicatum*, MUT4330, was shown to harbor six distinct proposed virus species including a fusari-like virus, Penicillium aurantiogriseum fusari-like virus 1 (PaFV1), a *Totivirus*, Penicillium aurantiogriseum totivirus 1 (PaTV1), a +stranded RNA virus named Penicillium aurantiogriseum foetidus-like virus 1 (PaFlV1) due to its similarity to *Aspergillus foetidus slow virus 2*, a bipartite virus named Penicillium aurantiogriseum bipartite virus 1 (PaBV1), a *Partitivirus*, Penicillium aurantiogriseum partitivirus 1 (PaPV1), and a virus with low similarity to the family *Partitiviridae* named Penicillium aurantiogriseum partiti-like virus 1 (PaPlV1). With a simple protocol we were able to isolate and enrich for particles of several viruses belonging to different taxonomic clades and with both dsRNA (4 viruses) and (+) ssRNA genomes (2 viruses); four viruses encode for a CP and are therefore purified as virions (PaTV1, PaBV1, PaPV1 and PaPlV1); a fifth ssRNA virus (PaFlV1) is likely encapsidated by PaTV1, as it is the case of the Yado-kari virus^22^.

From an evolutionary perspective, two are the requirements for a fungal virus to become a “bona fide” plant virus: i) the virus must be able to replicate in plant cells, and ii) the virus must acquire the ability to infect systemically the host plant. Systemic infection relies fundamentally on two strategies: ability to infect meristematic tissue (a strategy employed by cryptoviruses) or acquisition of a movement protein (a strategy employed by most plant viruses). Here we will test the first step of this evolutionary scenario, i.e. ability to infect plant cells. In order to avoid the movement components, we choose to work with plant protoplast systems, and in specific we choose *Nicotiana benthamiana* because it is the model plant for virus-host interaction. *N. benthamiana* displays a well-studied general susceptibility to plant virus infections^23–25^ that makes it the ideal species for our experimental setup. The second experimental system to test mycovirus replication is based on protoplasts derived from *N. tabacum* bright yellow 2 (BY2) cell line^26, 27^, a very reliable plant cell system that can be maintained indefinitely and from which protoplast can be easily obtained in axenic conditions and monitored for virus accumulation for up to 6–7 days.

We are here reporting the first evidence that two “bona fide” mycoviruses replicate in protoplasts of two different plant hosts, *N. benthamiana* and *N. tabacum* BY2 cell line.

## Results

### PaTV1 can replicate in WT *N. benthamiana* protoplasts

In order to test if mycoviruses can replicate in plant cells, we decided to enrich and partially purify virus particles from a single monoconidial isolate of *P. aurantiogriseum* (isolate MUT4330) and transfect them to plant protoplasts. We decided to perform our experiment using protoplasts from *N. benthamiana*, a model plant for virus-host interaction, due to its wide susceptibility to plant virus infection^24, 25^. In each experiment we analyzed three different transfection events for each time point of a time course. The T0 time point is representative of the virus RNA associated to the protoplast at the time of inocula (internalized or stably attached to the membrane after two washes). The T48 time point is the upper time limit for *N. benthamiana* protoplasts before a dramatic decrease in cell viability is observed (not shown). We decided to use the same T48 time point also for BY2 cells, in order to compare directly the two systems.

The partially purified virus suspension was filtered through a 0.22 μm mesh filter before transfection and an aliquot of the suspension was layered on fungal culture media in order to rule out the presence of contaminating mycelia in the protoplasts.

**Figure 1:**
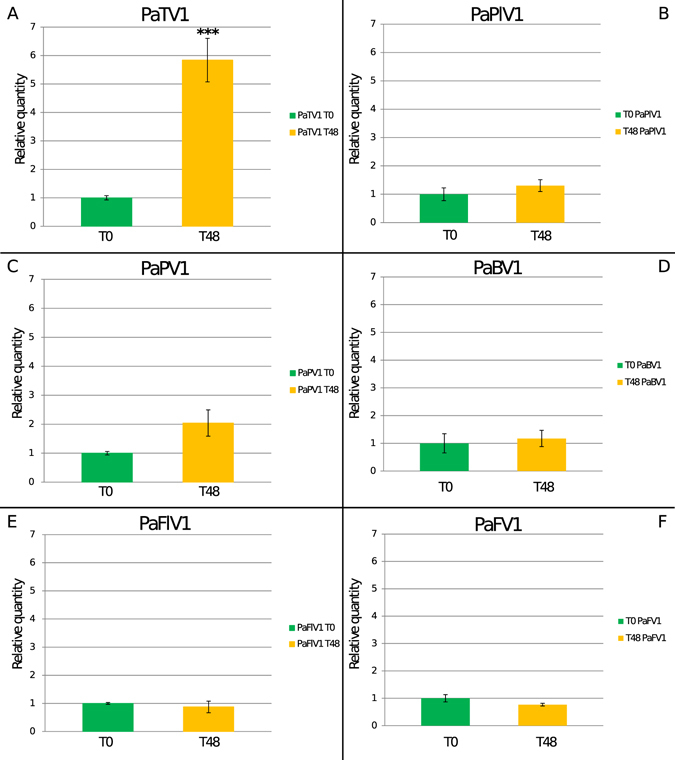
Absolute quantification of viral RNA in *Nicotiana benthamiana* protoplasts at T0 and T48 time points. Quantitative Reverse transcriptase PCR (qRT-PCR) data were used. We present the data as a fold change of viral RNA at T48 compared to T0 to which we arbitrarily assigned the value 1. In panel A absolute quantification of the Totivirus Penicillium aurantiogriseum totivirus 1 (PaTV1): quantification shows ca. 5 times more RNA at T48 (yellow bar) than T0 (green bar). In panels B, C, D, F and E quantification of the absolute virus RNAs shows no statistically significant difference over time. *P < 0.05 **P < 0.01 and ***P < 0.001.

Statistical analysis (Supplementary Table 1) of viral RNA accumulation through quantitative reverse transcriptase PCR (qRT-PCR), showed that in *N. benthamiana* WT protoplasts the only virus able to increase the amount of RNA in absolute terms between the T0 time point and the T48 time point is the totivirus PaTV1 (Fig. 1 and Supplementary Fig. 1). Quantification data showed over 5 times more RNA at T48 than at T0 (average of three biological replicates). All the other viruses showed no statistically significant increase, or decrease in RNA amount (Fig. 1 and supplementary Fig. 1) strengthening the idea of the specificity of the observed increased accumulation for PaTV1.

Maintenance of a relatively high level of viral RNA in transfected plant protoplast 48 hours after transfection could be due to protection from RNAse degradation provided by the virion (from their own genome encoded coat proteins for PaPlV1 and PaBV1 or from trans-encapsidation for the ssRNA virus PaFlV1, as is the case of the Yado-kari virus 1^22^). Another ssRNA virus (PaFV1) shows also limited decrease in RNA accumulation, likely because of its protection from anti-viral RNAse activity in host derived membranes, as it is the case of other members of the *Hypoviridae* family^28^.

The internal control *COX* ^29^ gene showed a typical decrease in amount due to protoplast senescence and death (not shown).

### *N. benthamiana* HC-Pro protoplasts support higher level of replication of PaTV1 and display evidence of replication of another mycovirus species

**Figure 2:**
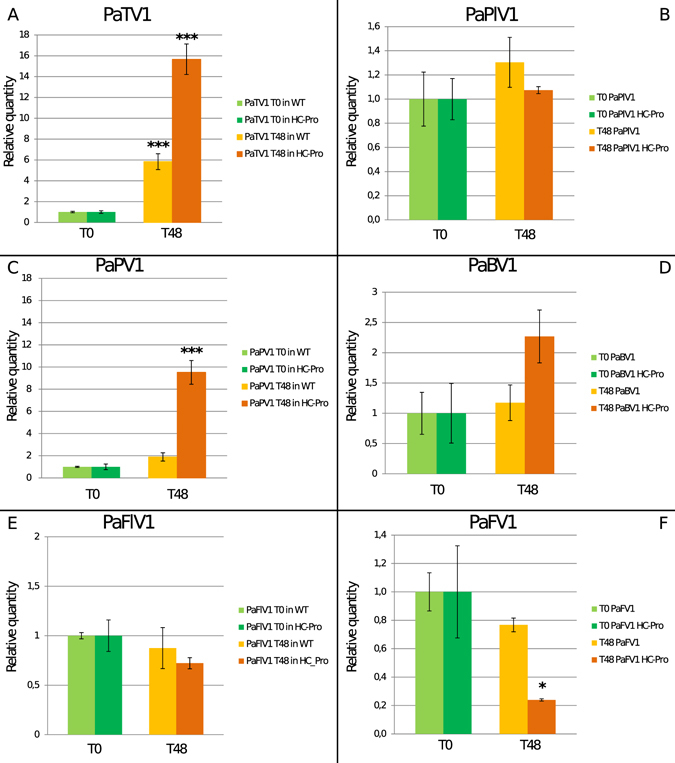
Comparison of virus accumulation in protoplasts from wild type (WT) and HC-Pro *Nicotiana benthamiana*. Quantitative Reverse transcriptase PCR (qRT-PCR) data from WT and HC-Pro *N. benthamiana* protoplasts were used to calculate fold change of viral RNAs at T0 and T48 time points. In panels A data for the Totivirus Penicillium aurantiogriseum totivirus 1 (PaTV1) are reported: for WT protoplasts we observed ca. 5 times more RNA at T48 (yellow bar) time point and ca. 15 times more RNA in HC-Pro protoplasts (orange bar). In panels C quantification of the Partitivirus Penicillium aurantiogriseum partitivirus 1 (PaPV1): no statistically significant differences were observed for WT protoplasts, but ca. 10 times more viral RNA was observed in case of HC-Pro protoplasts. Panels B, D, E and F show data for the other viruses: none of them displays a statistically significant increase in virus RNA load. *P < 0.05 **P < 0.01 and ***P < 0.001.

Antiviral defense in plants and fungi was shown to rely on the RNA silencing pathway and most viruses have the ability to suppress such pathway encoding a specific protein intended to interfere with the silencing process^30^: a further evidence of true viral replication would come from showing that in the presence of a silencing suppressor, virus accumulation increases specifically^31^. For this purpose, protoplasts for transfection were derived from a transgenic line of *N. benthamiana* expressing a silencing suppressor (HC-Pro) from *Turnip mosaic virus* (TuMV) shown to be able to enhance plant viral replication^32^. In this experiment we transfected side by side WT and HC-Pro protoplasts using the same virus preparation and then compared results. As previously observed, in WT plants the absolute RNA amount of PaTV1 was 5 times higher at T48 than at T0. By contrast, in HC-Pro protoplasts the amount was 15 to 20 times higher than at T0 (statistically different from absolute amount at T0, P value 0.000140) (Fig. 2 and Supplementary Fig. 2). Interestingly in HC-Pro plant protoplasts we observed also an increase in the amount of another virus, the fungal partitivirus PaPV1 which was 10 times higher 48 hours post transfection (statistically different from absolute amount at T0, P value 0.00609) (Fig. 2 and Supplementary Fig. 2); on the contrary, the other four viruses did not show evidence of a statistically supported increase in viral RNA accumulation over time (Fig. 2 and Supplementary Fig. 2).

### Both PaTV1 and PaPV1 can also replicate in BY2 tobacco cells

**Figure 3:**
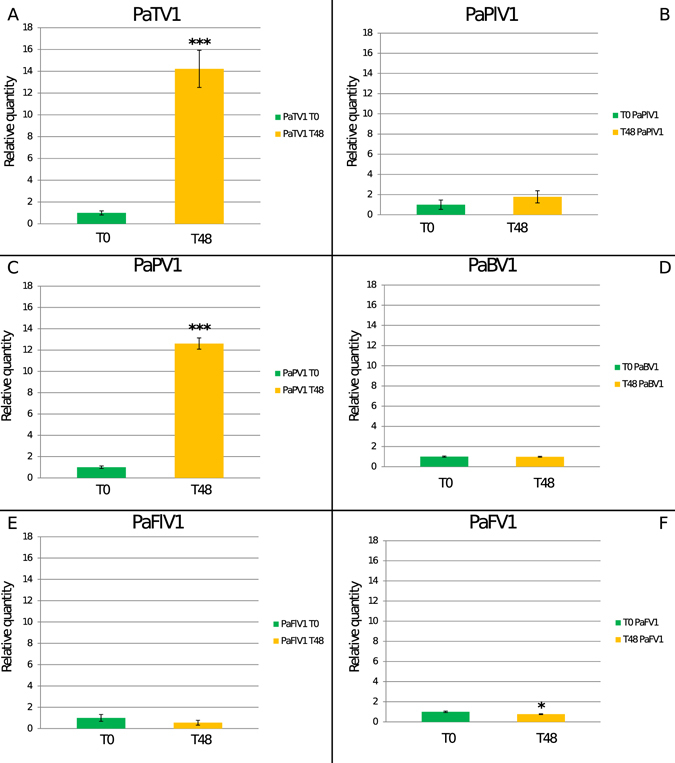
Absolute quantification of viral RNA in *Nicotiana tabacum* BY2 protoplasts at T0 and T48 time points. Quantitative Reverse transcriptase PCR (qRT-PCR) data were used. We present the data as a fold change of viral RNA at T48 compared to T0 to which we arbitrarily assigned the value 1. In panel A: absolute quantification of the Totivirus Penicillium aurantiogriseum totivirus 1 (PaTV1) quantification show ca. 14 times more RNA at T48 (yellow bar) than at T0 (green bar). In panels C: quantification of the Partitivirus Penicillium aurantiogriseum partitivirus 1 (PaPV1) show ca. 12 times more viral RNA at T48. Panels B,D,E and F show data for the other viruses: none of them displays a statistically significant increase in virus RNA load. *P < 0.05 **P < 0.01 and ***P < 0.001.

Due to natural specific conditions that usually confer ability to replicate in some hosts better than in others (virus specificity) we decided to test also tobacco BY2 cells to compare to the data observed using *N. benthamiana* protoplasts, and to determine if there is a different response in a different plant host. Real-time PCR data from BY2 cells confirmed viral replication also in these host cells. We observed 15 times more RNA at time 48 for PaTV1. Surprisingly in this case we observed also 10 times more RNA for the fungal PaPV1, but no evidence of replication for the remaining four viral species (Fig. 3 and supplementary Fig. 3).

### Mycovirus thermal inactivation

**Figure 4:**
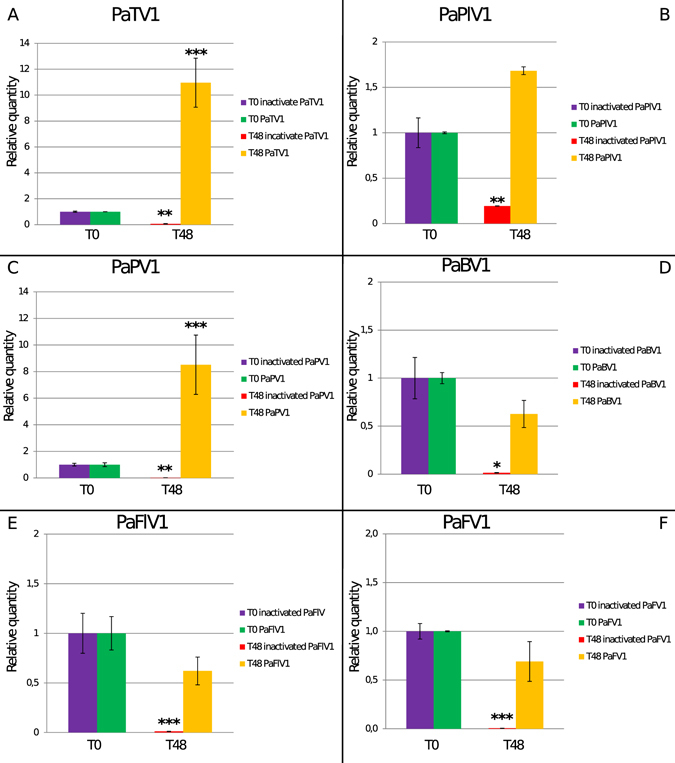
Comparison of virus accumulation in protoplasts of Nicotiana tabacum BY2 cells with filtered untreated or heat inactivated virus inocula. Quantitative Reverse transcriptase PCR (qRT-PCR) data from *Nicotiana tabacum* BY2 cells were used to obtain absolute quantification of viral RNA at T0 and T48 time points with heat-inactivated (purple for T0 and red for T48) or untreated (green for T0, yellow for T48) viral inocula. In Panel A: Quantification graph of PaTV1 confirmed the ability to replicate when the viral purification is untreated, whereas when inactivated before transfection the final RNA amount at T48 (red bar) was lower than at T0. In panels C quantification of the Partitivirus Penicillium aurantiogriseum partitivirus 1 (PaPV1): similarly to PaTV1, PaPV1 can replicate in untreated biological replicates whereas the RNA accumulation level decreased when thermally inactivated suspension was used for transfection. Panels B,D,E and F show data for the other viruses: none of them displays a statistically significant increase in virus RNA load. *P < 0.05 **P < 0.01 and ***P < 0.001.

**Figure 5:**
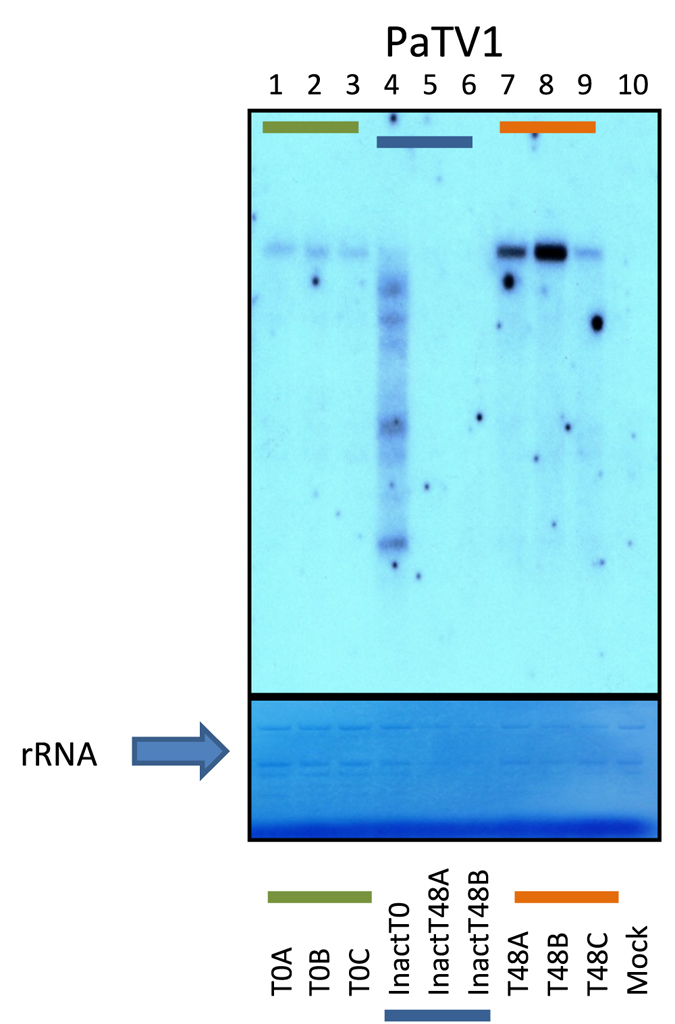
Northern blot analysis of total RNAs extracted from WT *Nicotiana benthamiana* protoplasts. A riboprobe was used to detect Penicillium aurantiogriseum totivirus 1 (PaTV1). The first three columns (green line, biological replicates T0_A_, T0_B_ and T0_C_) show lanes loaded with total RNA from T0 transfected protoplasts. Columns 7 to 9 (orange line, biological replicates T48_A_, T48_B_ and T48_C_) show lanes loaded with total RNA extractions of three more replicates transfected with untreated virus inocula harvested 48 hours after transfection. Columns 4 to 6 (blue line, biological replicates inactT0, inactT48A, inactT48B) show lanes loaded with total RNA for protoplasts transfected with inactivated viral purification: column 4, total RNA from viral inactivated transfected protoplasts at T0 showing clear signs of degradation of viral RNA (lack of a full length genomic RNA, and a number of lower molecular weight bands). In columns 5 and 6, total RNA from viral inactivated transfected protoplasts at T48, the viral genomic RNA and derivatives are completely cleared. As visible in methylene blue staining rRNA (arrow) amount was lower at T48 than at T0 in all samples, but the viral amount is higher only in untreated T48 protoplasts. Column 10 show mock inoculated protoplasts as negative control. The northern blot represent the full membrane; the lower panel represent the same membrane stained with methylene blue and cropped in the lower and upper part to show only the stained ribosomal RNA.

To confirm the above results and include another negative control, we repeated the experiments and compared transfection using thermally-inactivated virus inoculum (boiled for 5 minutes) with untreated inoculum in *N. benthamiana* WT and *N. tabacum* BY2 protoplasts. For both experiments we observed replication for PaTV1 only in protoplasts transfected with the untreated viral inocula (Fig. 4 and supplementary Fig. 4). By contrast, protoplasts transfected with thermally-inactivated inocula showed a decrease of RNA for PaTV1 (all the viruses are statistically lower at T48, P values in Supplementary Table 1). *N. benthamiana* transfection data were confirmed by northern blot assay (Fig. 5) which showed that the three replicates at the T48 time point displayed higher accumulation than the T0 time point in the untreated purified virus transfections. On the contrary, thermally-inactivated transfected inocula resulted in i) higher amounts of total viral RNA (encapsidated genomic RNA and replicative forms) from the transfected protoplasts at T0, and ii) total degradation of the full length genomic RNA at T48 (Fig. 5).

### Cryphonectria hypovirus 1 does not replicate in BY2 cells

Among the six virus species present in the fungal isolate used as source for virus inoculum, one species, PaFV1, a fusari-like virus closely related to the *Hypoviridae* family – a family of capsid-less viruses-, could only partially be concentrated with the protocol we used (more oriented to virus species possessing true virions). For this reason we decided to test if another virus belonging to the *Hypoviridae* family, and to the species *Cryphonectria hypovirus 1* (CHV1), was able to replicate in BY2 cells: in this case we could bypass the inefficiency of our concentration protocol because we can take advantage of an infectious cDNA clone that can be used for the synthesis *in vitro* of full length viral RNA^33^.

Real time PCR data clearly show that CHV1 was not able to replicate in BY2 cells bringing to complete clearance of the infectious transcript in 48 hours (Supplementary Fig. 5). As a control for infectivity of the transcripts, the same RNA was used on *C. parasitica* protoplasts, and resulted in CHV1 infected *C. parasitica* isolates (not shown).

### Molecular analysis of virus populations in protoplasts transfections

We next performed re-sequencing of PaTV1 and PaPV1 genomes present in plant protoplasts at 48 hours after transfection. This was done by RT-PCR with specific primers thorough genome walking of overlapping segments in order to verify if the viral genome changed to adapt to the new host. The consensus sequences observed for both viruses were exactly the same to those previously determined through NGS in their fungal host^2^.

## Discussion

The diversity of mycoviruses was unknown or underestimated for many years and their importance in virus evolution has not been properly considered until recently^34^. At the same time, for many years mycoviruses were believed to have in nature narrow host ranges with abilities only to infect the same or closely related natural vegetative compatibility groups of the same fungal species^35^. Wider host ranges were experimentally obtained for CHV1 and Rosellinia necatrix victorivirus 1 (RnVV1)^36, 37^. However, in a few cases evidence of wider natural host ranges were reported: Botrytis porri virus 1 (BpRV1), a bipartite dsRNA mycovirus, originally detected in *Botrytis porri* was also found in *B. squamosa* and in *S. sclerotiorum* ^38^. Because of the quasispecies nature of virus infection in an individual host, virus variants can quickly adapt to new environments^39^. For this reason, in the last few years, the classic hypothesis of a long coevolution history between mycoviruses and fungi is now flanked by the hypothesis that viruses can move easily from fungal host to new hosts belonging to unrelated (distant) taxonomic clades^4^. This latter hypothesis seems to fit cases in which fungi are in close physical relationship with other organisms, such as is the case of fungal-plant interface for plant pathogenic or plant endophytic fungi. In our case we can envision the possibility for a mycovirus associated to *Posidonia oceanica* endophytes to become a plant virus: nevertheless previous small RNA characterization from *P. oceanica* plants failed to reveal any virus infection^2^. Certainly evolutionary and phylogenetic analysis support viruses changing and adapting to new types of hosts. One recent example is that of a new mycovirus with a genomic sequence related to Hepatitis virus E which was reported from *Sclerotinia sclerotiorum* ^40^, a plant pathogen. This suggests the possibility for inter-kingdom host jumps that can occur not only from fungi to plants and vice versa, but also from fungi to animals and vice versa.

A new evolutionary model that linked plant and fungal viruses was first proposed based on studies on the Cryphonectria hypovirus complex (CHV1, CHV2 and CHV3) which showed phylogenetic relationships of these viruses with plant-infecting viruses of the genus *Potyvirus* ^8^. Failure of CHV1 to replicate in plant cells can be explained by the fact that although it was the first mycovirus for which a phylogenetic link with plant viruses was shown, such a link is fairly distant, and in fact, the hypovirus-like clade does not harbor a mosaic of plant and fungal hosts, as is the case of the families *Partitiviridae* and *Totiviridae* ^41^.

Our report is the first to show experimentally that some mycoviruses can replicate in plant cells. We used three different plant protoplast systems to demonstrate replication and our results confirmed replication for two mycoviruses. We want to specify that all the data used in this paper come from absolute quantification of RNA in total RNA extractions, comparing the amounts at T0 and at T48 of exactly the same amounts of protoplasts transfected with the same amount of virus inocula: we did not perform any adjustment of RNA quantities or make relative quantification in relation to an endogenous control gene. When we used the 2^−ΔΔCt^ approach^42^ the fold change of replicating viruses was greater than considering absolute quantity (Supplementary Fig. 6). Nevertheless we wanted to use the more conservative method, since the different degradation kinetics for a mRNA compared to a virus dsRNA could in theory result in higher relative detection of the mycovirus RNA that is not due to virus replication, but to faster turnover (degradation) of the endogenous control mRNA. Furthermore, other methods relying on detection of increased negative strand accumulation, are not suitable since most of the viruses used in our experimental system have dsRNA genomes. None of the viruses we tested expresses genetic information through accumulation of subgenomic RNAs (an expression strategy almost absent in mycoviruses), therefore this approach, which is often used to demonstrate replication, could not be used. Therefore, we pursued the idea of simply comparing the amount of total virus RNA at T0 compared to the total amount of viral RNA at T48.

The totivirus PaTV1 showed replication differences between *N. benthamiana* WT and transgenic *N. benthamiana* expressing HC-Pro, a potyviral helper component proteinase suppressor of antiviral RNA silencing, where the increase of viral RNA was much greater. In both cell types we observed PaTV1 replication (absolute RNA amount was higher after 48 hours) but in case of HC-Pro cells the final concentration was at least twice that present in WT cells. This is a further indication of viral replication, because, as expected, in presence of the HC-Pro RNAi suppressor, accumulation was greater. Moreover increase of the partitivirus PaPV1 RNA in HC-Pro protoplasts but not in WT protoplasts further confirmed replication. In fact active RNA silencing machinery present in WT plants can be the limit for potential viral replication preventing increase in viral RNA amount, becoming possible instead in the absence of this defense mechanism. Interestingly both mycoviruses replicating in plant protoplasts belong to two families, *Partitiviridae* and *Totiviridae*, that show a mix of plants and fungal hosts^43, 44^. The two mycoviruses replicating in plant cells have both dsRNA genomes, and are encapsidated in isodiametric virions. Both genomes encode only for two proteins (the CP and the RdRp) in two distinct genome segments (for the partitivirus PaPV1) or in one genome segment through frameshift (for the totivirus PaTV1).

An interesting observation comes from the results observed in BY2 transfected cells. Comparing the ratio of initial and final amount of the *Totivirus* PaTV1 RNA, this virus seems to have a similar capability to replicate in *N. benthamiana* and *N. tabacum* cells. On the other hand the *Partitivirus* PaPV1, able to replicate only in HC-Pro but not in WT *N. benthamiana* cells, showed a good increase in RNA accumulation in BY2 cells. This result can be explained by host specificity. Both cell types come from plants of the genus *Nicotiana*, but they are different species and host specific components required for replication could play a role in host-specific response^45^.

Future studies on ability of mycoviruses to replicate in plants could entail whole organism experimental systems complementing the absence of a movement protein (MP) in mycoviruses: this approach was taken for Flock House virus (FHV), a (+)ssRNA insect virus: transgenic line of *N. benthamiana* expressing red clover necrotic mosaic virus MP or tobacco mosaic virus MP complemented the ability of FHV to move systemically in these plants^46^. This experimental approach is supported also from the evolutionary point of view at least in the case of ourmiaviruses: this taxonomic group displays a chimeric genome, generated by the reassortment of at least two distinct viruses; the proposed hypothesis for the origin of this family is that a capsid-less narnavirus of a plant associated fungus became a plant virus acquiring movement protein (MP), and coat protein (CP) from other plant viruses, most likely from members of *Tombusviridae* ^10^.

Recent work on the characterization of the viromes of a number of invertebrate phyla deeply changed our understanding of the RNA virosphere^4^. With the report of more than 1400 new viruses, some of which are from host taxa previously unexplored, almost all the viral families that we previously thought to be host-specific are now showing a mosaic of hosts belonging to distant taxonomic clades suggesting that strict virus-host co-divergence cannot always be assumed. Clear example of viral cross-species transmission between different taxa was proposed for viruses of plants and arthropods^4^. Here in this work we reported for the first time the experimental evidence that these events can occur also for mycoviruses.

## Materials and Methods

### Fungal growth and viral purification

*P. aurantiogriseum* var. *viridicatum* (MUT4330)^20^ was maintained on solid CYA prepared as previously described^2^. Virus inoculum was prepared by concentrating virus particle from fungus grown in liquid media, harvested after 72 hours of rotary shaking at 120 rpm and lyophilized, as previously described^2^. Inoculum was passed through a 0.22 µm filter to avoid any contamination of the virus suspension by fungal hyphae or conidia. At the time of transfection, an aliquot of the virus suspension was deposited on CYA solid media in order to rule out any fungal contamination post filtration.

For CHV1, infectious RNA was obtained *in vitro* through T7 transcription of linearized plasmid pLDST as previously described^33^ and ability to infect was tested on *Cryphonectria parasitica* protoplasts.

### Plant tissue

*N. benthamiana* seeds, WT or HC-Pro, were sterilized by washing in 70% ethanol for 5 minutes first and then 5% NaClO and Tween 20 0.1% for 15 minutes. Seeds were then washed 10 times with sterile water and sowed in Magenta boxes containing MS organic 0.44%, sucrose 1.5% and agar 0.7% supplemented with light 16 hours per day.

BY2 cells were maintained in modified KMCS media (0.44% MS minimal salts, 3% sucrose, 1 mg/l thiamine, 50 mg/l *myo*-inositol, 0.2 mg/l 2–4 D) on a rotary shaker at 120 rpm and refreshed in new media with a ratio of 1/10 every 72 hours.

### Protoplasts preparation and virus transfection

Forty days after sowing, *N. benthamiana* leaves were harvested from plants, rubbed with Q-tip soaked in sterile 1% celite and 0.25 M potassium phosphate pH 7 and placed in a sterile petri dish containing 20 ml of Mannitol 10%, Cellulysin (Calbiochem 219466) 1.25%, Macerozyme (MP Biomedicals 152340) 0.125% and Bovine serum albumin (BSA) 0.125%. Petri dishes containing leaves floating on the digestion mix were gently shaken at 65 rpm for 4 to 5 hours. Cells were sedimented in Corex tube by centrifuging 6 minutes at 100 g. Protoplast were then washed twice with 10% mannitol and then cleaned from cells debris on a 20% sucrose cushion. Protoplasts were counted and adjusted to 3 × 10^6^/ml.

For each transfection event 1 ml of protoplast suspension was distributed in sterile glass tubes and sedimented at 100 g for 3 minutes. Protoplasts were then resuspended in 20 µl of inoculum, then transfected with 100 µl of a solution of PEG1450 40% and CaCl_2_ 5%, shaken for 10 seconds and later mixed in 1 ml of ice cold 10% mannitol. Thirteen minutes later, protoplasts were sedimented and resuspended in 1 ml of AOKI solution (2 mM KH_2_PO_4_, 10 mM KNO_3_, 10 mM MgNO_3_, 100 mM CaCl_2_, 10 µM KI, 1 µM CuSO_4_.5H_2_O). Protoplasts harvested at T0 were sedimented again and stored at −80 °C; biological replicates harvested at T48 were sedimented and freezed at −80 °C before RNA extraction.

Protoplasts from BY2 cells were obtained from 72 hours subculturing in modified KMCS media. Cells were harvested in a 50 ml tube by centrifuged (100 g for 6 minutes), and then collected supernatant was conserved as conditioned medium; protoplasts were then washed in MM washing buffer (5 mM CaCl_2_, 12 mM Na-Acetate, 425 mM Mannitol, pH 5,8) in which the cells were left floating for at least 40 minutes. After that the supernatant was removed with a sterile pipette and replaced with 30 ml of enzyme solution (1% Cellulysin, 0.6% Cellulase, 0.4% Macerozyme, 5 mM CaCl2, 12 mM Na-Acetate, 370 mM Mannitol, pH 5,8). Cells were digested on a rotary shaker for 4 to 6 hours at 50 rpm. To collect protoplasts the cell suspension was passed through a sterile 70 µm nylon filter and collected in a 50 ml tube. Enzyme solution was removed by centrifugation (90 g for 5 minutes) and cell washed two times in MM wash solution. After the second washing step, cells were then resuspended in 20 ml of MM wash solution, counted in a Burker chamber and the concentration adjusted to 2 × 10^6^ cells/ml. One ml of protoplasts for each transfection event were then aliquoted in 15 ml polystyrene tubes, centrifuged at 90 g for 3 minutes and supernatant removed. Inoculum was added (20 µl) to the protoplasts which were then resuspended and transfected by adding 200 µl of sterile PEG solution (50% PEG1450, 15 mM MgCl_2_, 100 mM Ca(NO_3_)_2_). After 20 seconds of gently shaking, 1 ml of MM wash solution was added and protoplasts were kept on ice for at least 15 minutes. Transfection solution was then eliminated by centrifugation (90 g for 3 minutes) and 10 ml of filter sterilized culture media (5% coconut water, 20% conditioned, 265 mM mannitol and modified KCMS to volume) were added. Protoplasts were maintained in 60 mm petri dishes at 25 °C without light for 48 hours and then collected by centrifugation (90 g for 3 minutes).

Efficiency of transfection was estimated using purified virions TMV expressing GFP (from agroinfectious clone pJL24)^47^. In standard experiments for both *N. benthamiana* and BY2 protoplasts the rate of infected protoplasts was over 80%.

### Northern blot analysis

Total RNA was extracted from cells from each transfection event and then separated by denaturing agarose gel electrophoresis (glyoxal method) using HEPES-EDTA buffer^48^. A radio-labeled probe for PaTV was prepared trough T7 transcription from linearized plasmid as already described^2^.

### Real time PCR

RNA extractions were performed with Total Spectrum RNA Reagent (Sigma-Aldrich, Saint Louis, MO, USA) and RNA concentration was quantified with a NanoDrop 1000 Spectrophotometer. Copy DNA was obtained with High-Capacity cDNA Reverse Transcription Kit (Thermoscientific, Waltham, MA, USA), using 5 µl of total RNA without normalizing the amount between samples, in order to be able to check for absolute increase in virus accumulation among different time points. For each virus or gene analyzed in the three biological replicates we did three technical replicates in Real time PCR (list of specific primers in supplementary Table 2). Each experiment was repeated at least three times. Relative quantification was calculated using the 2^−ΔΔCt^ method^42^.

### Viruses re-sequencing from plant protoplasts

Specific primers designed on PaTV1 and PaPV1 sequences were used to obtain cDNA from protoplasts total RNA extraction with Invitrogen Thermo-Script SuperScript IV RT kit. Obtained cDNAs were used as template in a classic PCR protocol using specific primers (Supplementary Table 3), products were cloned in the pGEM-T easy vector (Promega, Madison, WI, USA) and sequenced by dideoxy chain termination method^49^ at BioFab Research (Rome, Italy). Information about each nucleotide position derives from at least 3 distinct clones.

### Statistical analysis

Threshold detection levels were calculated automatically by the Bio-Rad CFX Manager Software version 3.0 (Bio-Rad). When raw data were not normally distributed and variances were not homogeneous, they were log-transformed before analysis. The t-test was used to compare virus quantities measured at different sampling times. Statistical tests were performed with SIGMAPLOT 13 (Systat Software).

## Electronic supplementary material

Supplementary figures and tables
