# Supplementary material for: Mycoviruses of an endophytic fungus can replicate in plant cells: evolutionary implications
Source: Sci Rep. 2017 May 15;7:1908. doi: 10.1038/s41598-017-02017-3 (PMC5432518; doi:10.1038/s41598-017-02017-3)
Supplement: Supplementary file 1 — Supplementary figures and tables [file 41598_2017_2017_MOESM1_ESM.pdf]

Mycoviruses of an endophytic fungus can replicate in plant cells: evolutionary implications.

L. Nerva[1-2-3], G.C. Varese[2], B.W. Falk[3] and M. Turina[1*]

[1] *Institute for Sustainable Plant Protection, CNR, Strada delle Cacce 73, 10135 Torino, Italy*
[2] *Mycotheca Universitatis Taurinensis (MUT), Department of Life Sciences and Systems Biology, University of Turin, Viale Mattioli 25, 10125 Torino, Italy*
[3] *Plant Pathology Department, University of California Davis, Davis, CA 95616, USA*

Supplementary Figures and Tables

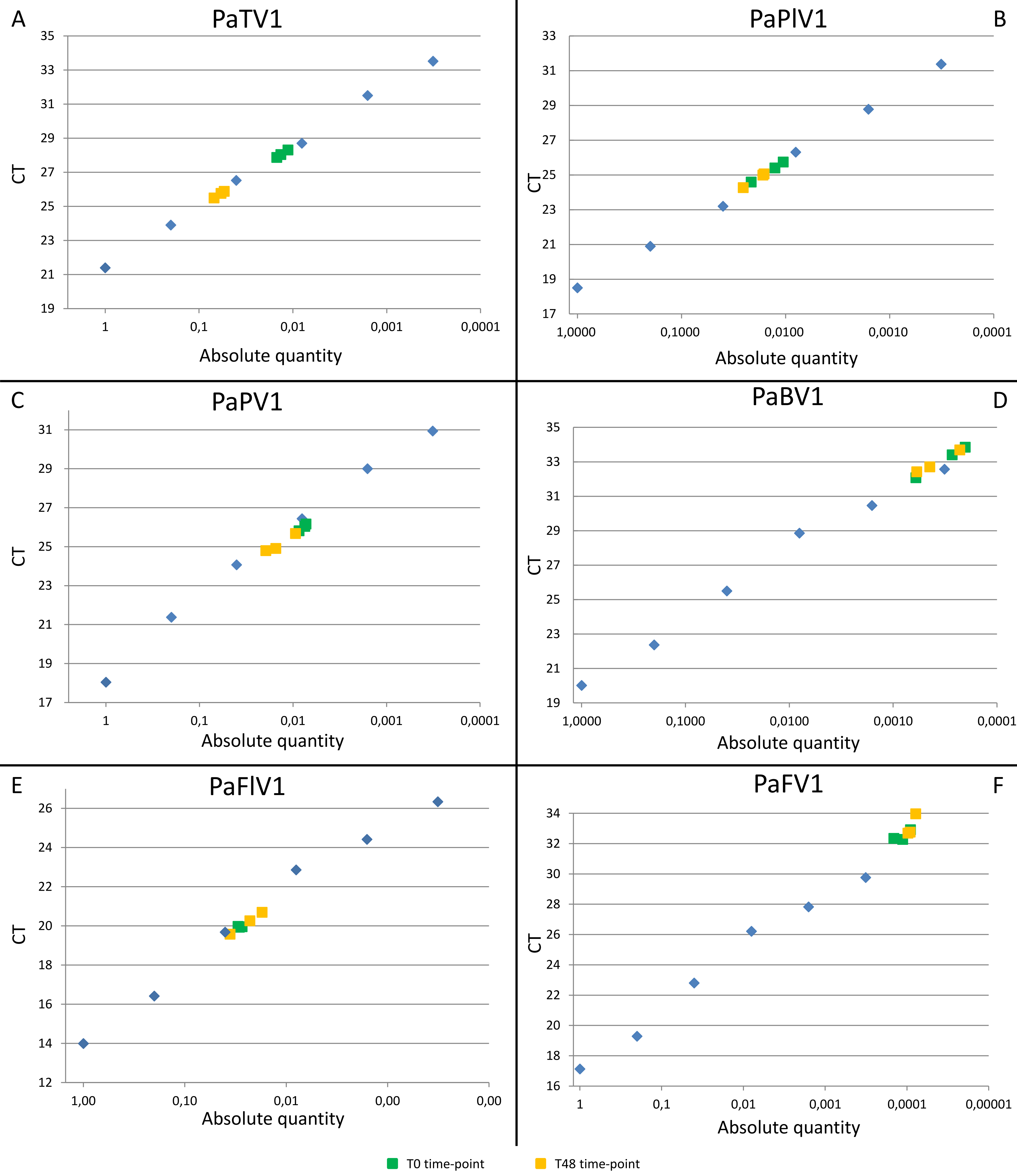

Supplementary Fig.1. Absolute quantification of transfected viruses at T0 and T48 time points in *Nicotiana benthamiana* protoplasts. Absolute quantifications (obtained from qRT-PCR) were plotted on standard serial dilution (1:5) curves for each virus. Panel A is Penicillium aurantiogriseum totivirus 1 (PaTV1) which shows two well distinguishable groups, T48 show an increase in PaTV1 amount. Panels B, C, D, E and F present data for the other viruses: they are not statistically different over time. Three biological replicates were done and value of each dot represents the mean of three technical replicates.

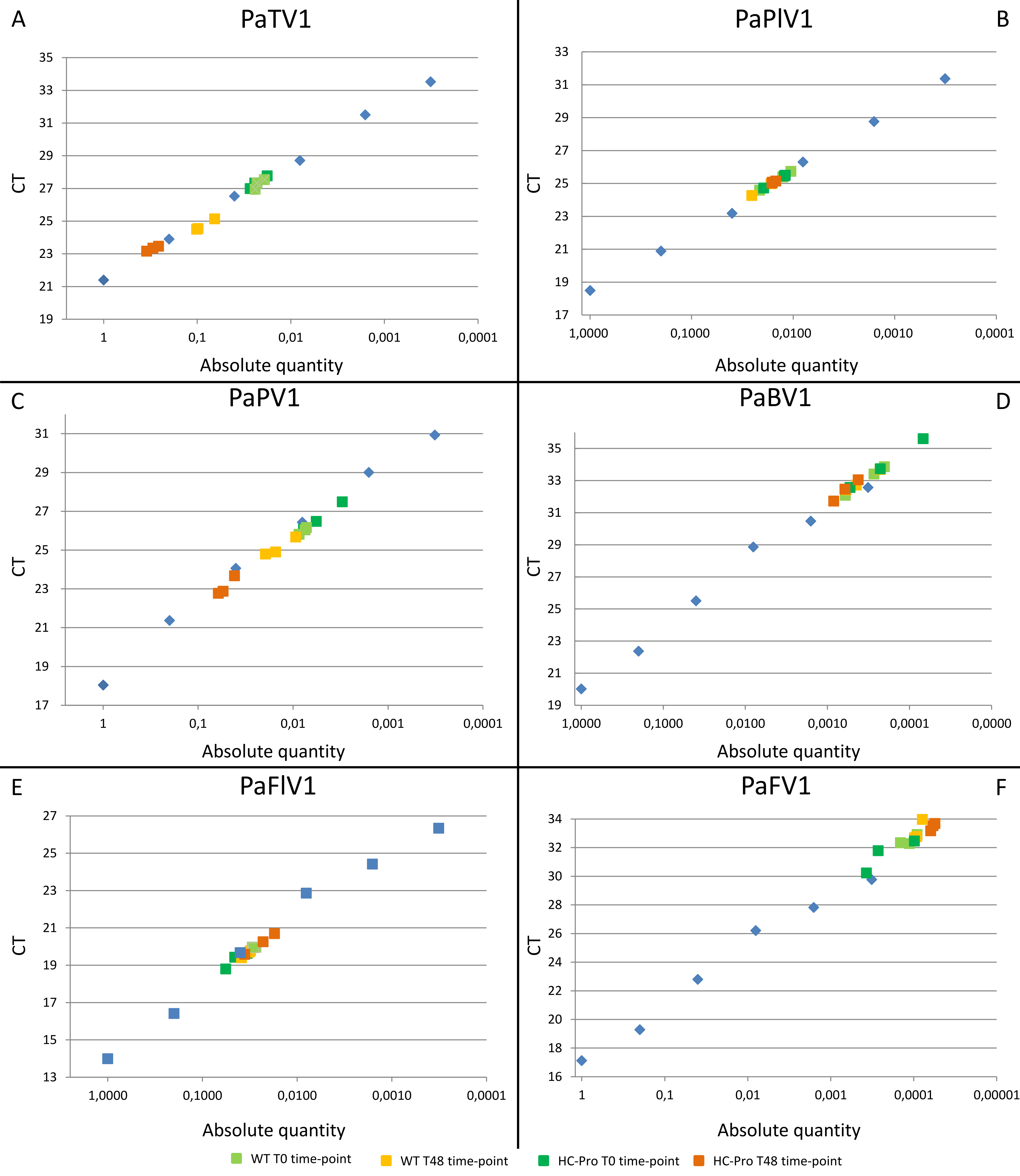

Supplementary Fig.2. Virus accumulation in WT and HC-Pro *Nicotiana benthamiana* protoplasts at T0 and T48 time points. Absolute quantifications (obtained from qRT-PCR) were plotted on serial dilution (1:5) standard curves for each virus. Panel A is Penicillium aurantiogriseum totivirus 1 (PaTV1) which show three well distinguishable groups showing an increase in viral RNA amount between T48 in *N. benthamiana* WT (yellow dots) and T48 *N. benthamiana* HC-Pro (orange dots). Panel C is Penicillium aurantiogriseum partitivirus 1 (PaPV1) which show increase of viral RNA only in HC-Pro protoplasts (orange dots). Panels B, D, E and F show the same data for the other viruses: non of them could replicate. Three biological replicates were done and value of each dot represents the mean of three technical replicates.

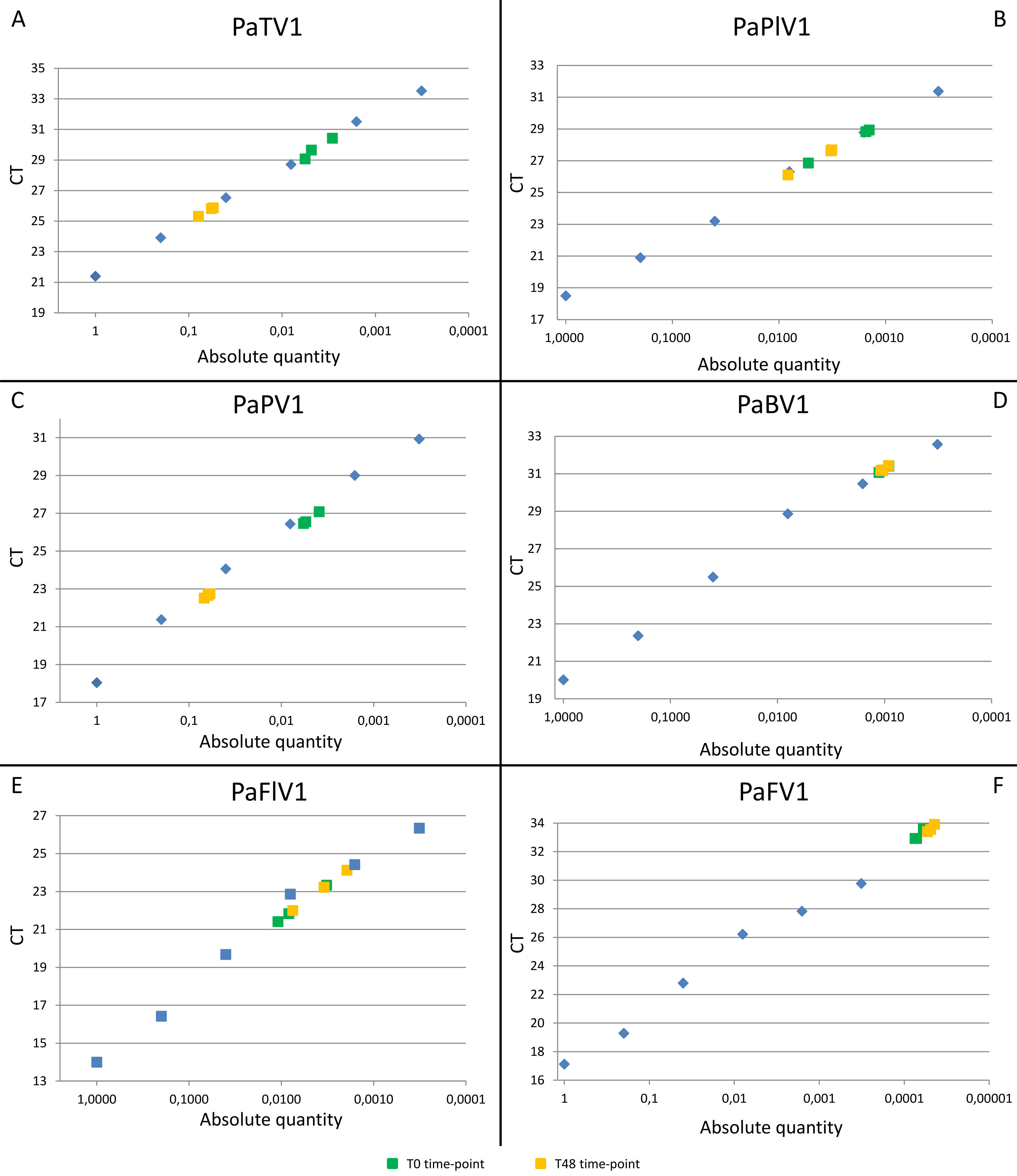

Supplementary Fig.3. Absolute virus RNA accumulation in transfected *Nicotiana tabacum* protoplasts at T0 and T48 time points. Data (obtained from qRT-PCR) were plotted on standard serial dilution (1:5) curves for each virus. Panel A is Penicillium aurantiogriseum totivirus 1 (PaTV1) which shows two well distinguishable groups, T48 (yellow dots) showing an increase in PaTV1 RNA amount. Panel C is Penicillium aurantiogriseum partitivirus 1 (PaPV1) which also shows an increase of viral RNA at T48. Panels B, D, E and F present data for the other viruses: they are not statistically different over time. Three biological replicates were done and value of each dot represents the mean of three technical replicates.

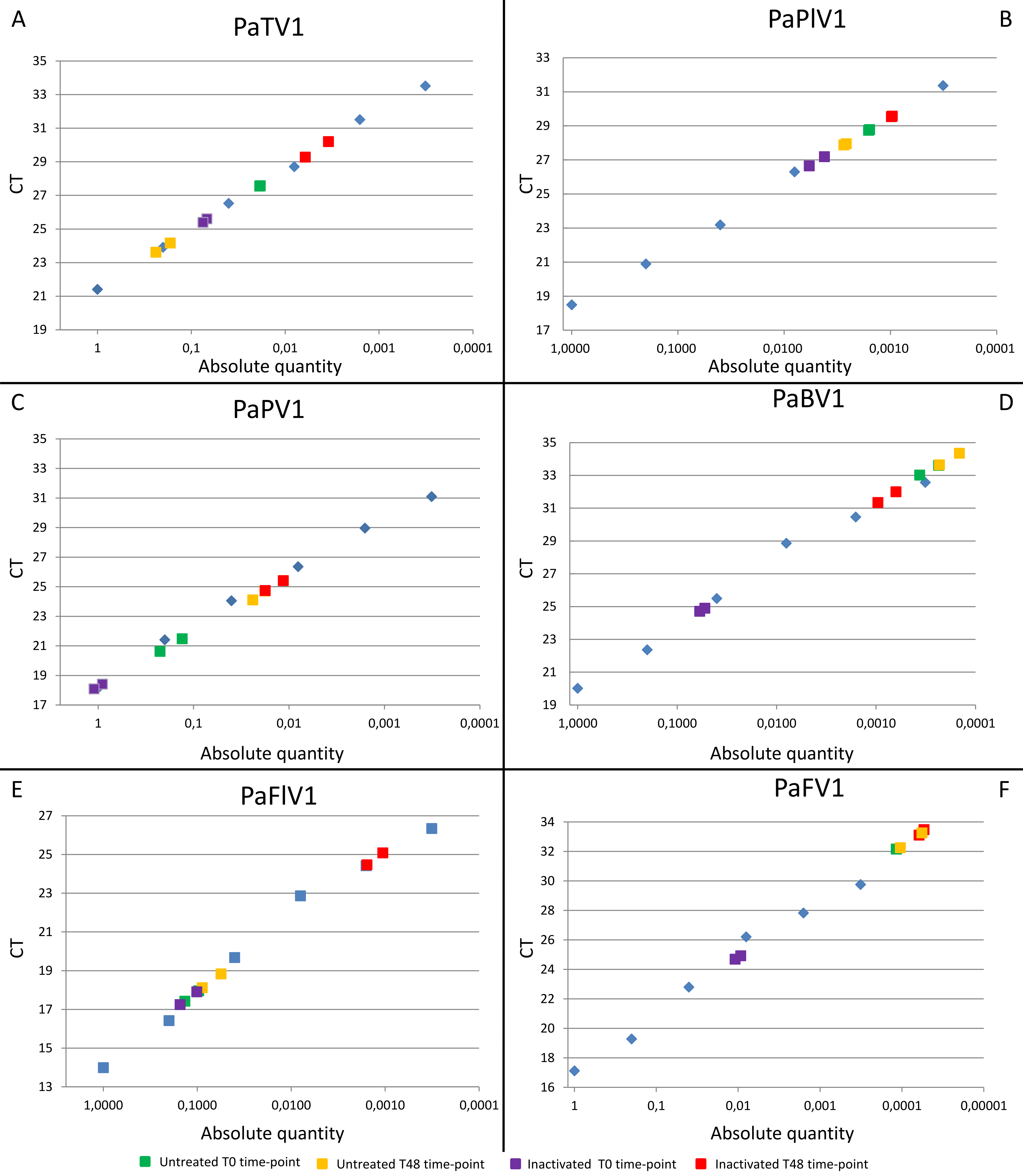

Supplementary Fig.4. Quantification of virus RNA at T0 and T48 time points for both inactivated or untreated viral inocula in *Nicotiana tabacum* BY2 cells protoplasts. Quantitative Reverse transcritpase PCR (qRT-PCR) data were plotted on serial standard dilution (1:5) curves for each virus. Panel A is Penicillium aurantiogriseum totivirus 1 (PaTV1) and panel C is Penicillium aurantiogriseum partitivirus 1 (PaPV1): they both show an increase in viral RNA amount only when the viral inocula are untreated. On the other hand they both show decrease when thermally inactivated inocula were used to transfect protoplasts. Panels B, D, E and F show data for the other viruses: quantification show a decrease over time independently if the viral purification was inactivated or not, with the exception of PaPlV1 for which there is no statistically difference between T0 and T48 transfected with untreated viral purification. Each dot represents the mean of three technical replicates.

CHV1

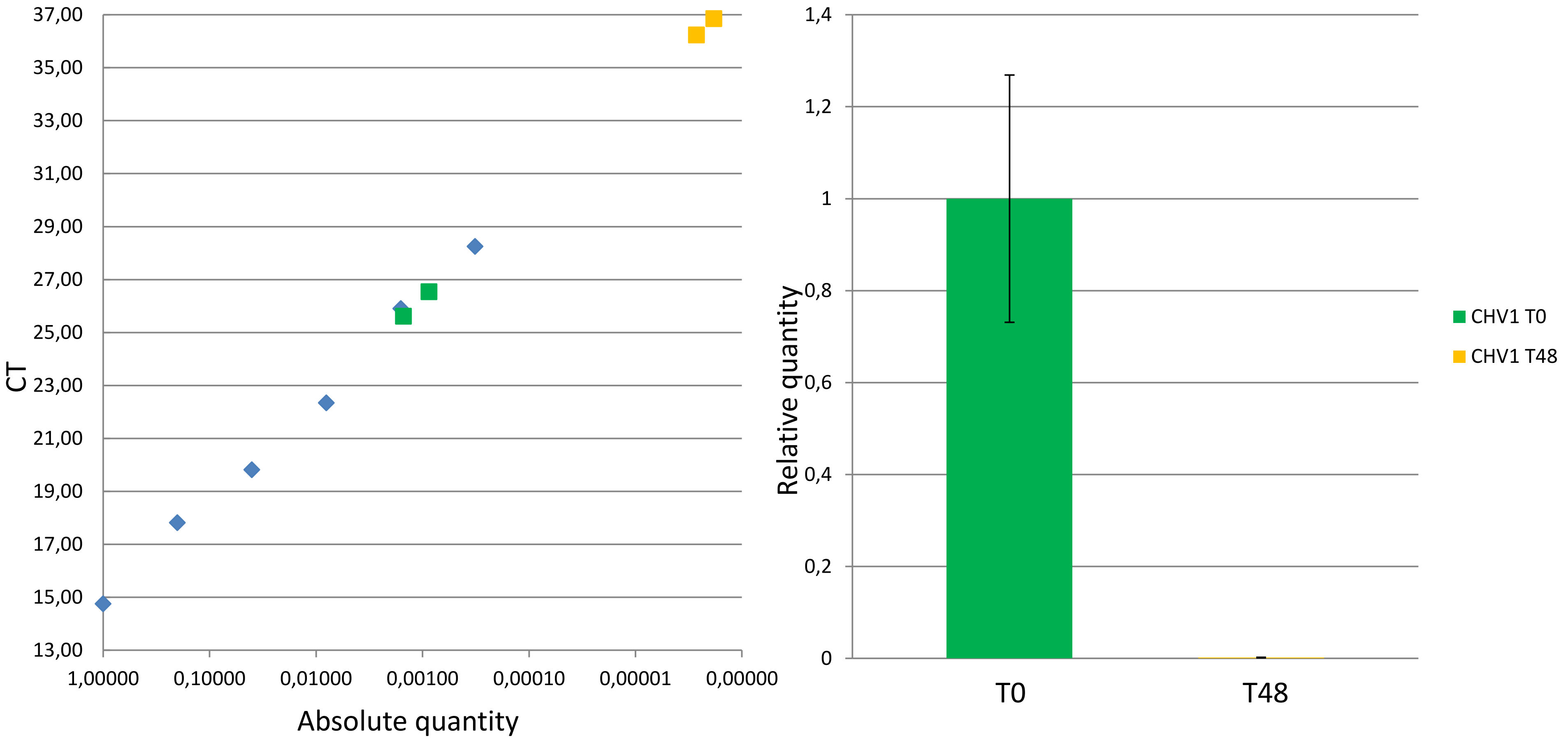

Supplementary Fig.5. Cryphonectria hypovirus 1 RNA accumulation at T0 and T48 time points in protoplasts derived from *Nicotiana tabacum* BY2 cells. Quantitative Reverse transcritpase PCR (qRT-PCR) data were plotted on serial dilution (1:5) standard curve. On the right side we present the data as a fold change of viral RNA at T48 compared to T0 to which we arbitrarily assigned the value 1. Two biological replicates were done and value of each dot represents the mean of three technical replicates. Green dots represent T0 time point and yellow dots represent T48 time point.

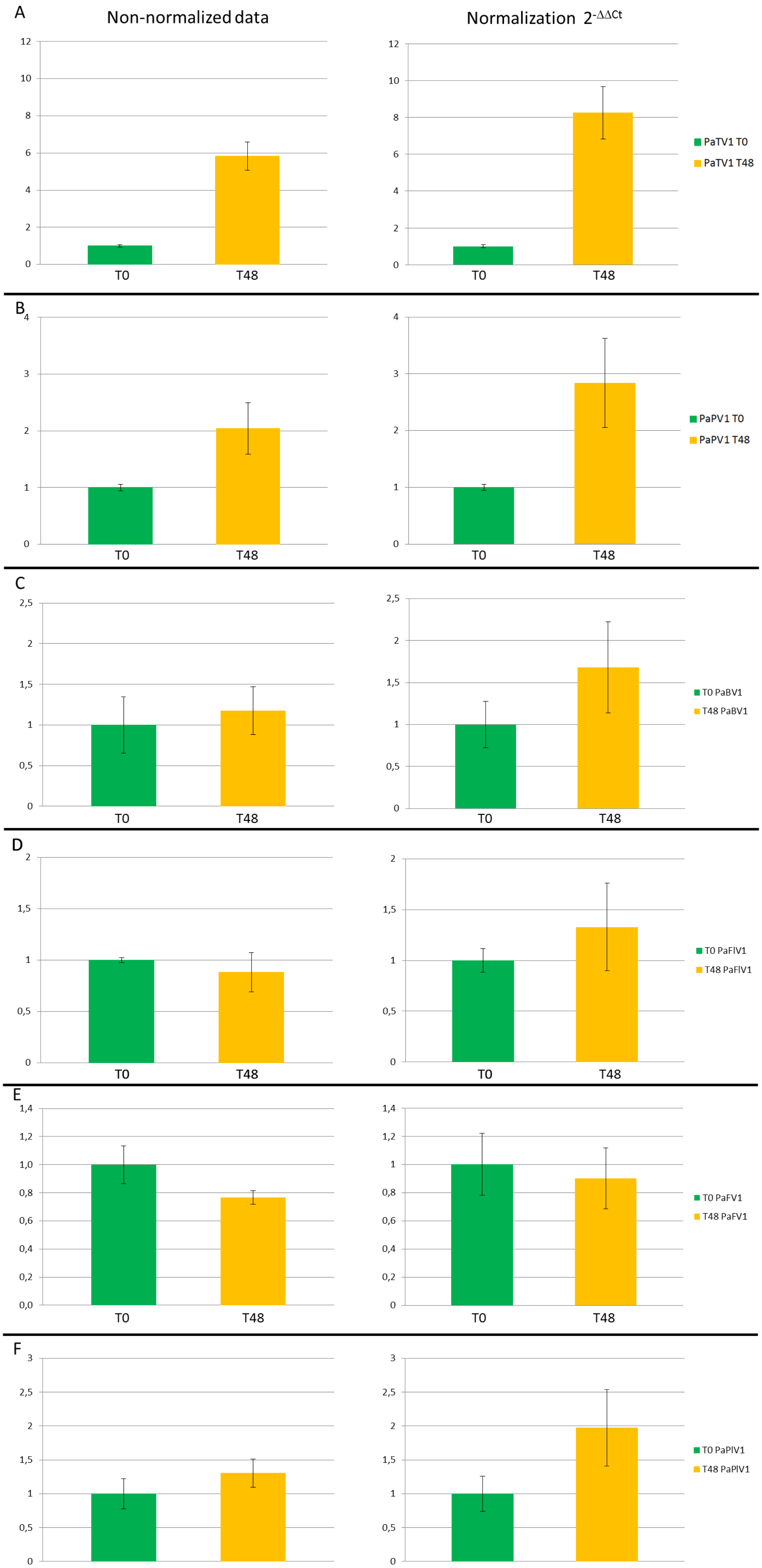

Supplementary Fig.6. Comparison of absolute and relative quantification methods for evaluating virus RNA accumulation. Quantitative Reverse transcritpase PCR (qRT-PCR) data from WT *Nicotiana benthamiana* protoplasts were used to calculate relative quantities (using COX as an internal control) with $2^{-\Delta\Delta Ct}$ method[40] and then compared with non-normalized data (no adjustment for internal control). In Panel A relative quantities were calculated for Penicillium aurantiogriseum totivirus 1 (PaTV1). With $2^{-\Delta\Delta Ct}$ method the average quantities at T48 result higher (8 times more than T0) than with non-normalized data (6 times more than T0). In panel B the same calculation was obtained for Penicillium aurantiogriseum partitivirus 1 (PaPV1). $2^{-\Delta\Delta Ct}$ normalization show a higher amount (3 times more than T0) than non-normalized data (2 times more than T0). The other viruses are reported in panels C, D, E and F and show a similar trend.

Supplementary Table 1. Summary of P values. P values were calculated on absolute quantities of three (or two in the case of inactivated viral purification) biological replicates at T0 and T48. P value < 0.01 means significant difference.

Protoplast Cell type	PaTV1	PaPV1	PaFlV1	PaBV1	PaPlV1	PaFV1
WT[a]	0.000153	0.0548	0.47	0.668	0.328	0.167
HC-Pro[a]	0.000069	0.00077	0.176	0.157	0.579	0.0325
BY2[b]	0.000331	3.84E-05	0.337	0.897	0.275	0.0192
BY2i[c]	0.0108	0.00322	0.0038	0.0498	0.0101	0.000427

[a] WT and HC-Pro *N. benthamiana* protoplasts transfected with viral suspension
[b] *N. tabacum* BY2 protoplasts transfected with viral suspension
[c] BY2 transfection with thermally inactivated virus

Supplementary Table 2: List of specific primers used for Real time PCR.

Virus	Nucleotide sequence
Penicillium aurantiogriseum fusarivirus 1	For: CGCGGTGCAGGAGAAGA<br>Rev: CCAAGACACACAACCTGAC
Penicillium aurantiogriseum totivirus 1	For: GAGGAGGCGACGGATCAA<br>Rev: CCTAGTCAGCGCCCTAGTGTATAAA<br>Probe: Fam-CCCGGGCTATCGGCCGACAG-Tamra
Penicillium aurantiogriseum asp-foetidus like virus	For: CAAGGTCGAGATAATTGCCGATA<br>Rev: TCTGGAGTCCCCTCTGGTCTATAC
Penicillium aurantiogriseum bipartite virus	For: CTCAACCTGTGGCTCTAACCAATC<br>Rev: ATATCGACGCAGCCGGTAAT
Penicillium aurantiogriseum partitivirus	For: CCTTAGGGTGCTGGGTGATG<br>Rev: CCTTGGCTTGTTCCAGACTGA
Penicillium aurantiogriseum partiti-like virus	For: GTCGCTACATCCCTGATCTCCTA<br>Rev: GTGTCTGTCACGAAAGCGAAAG<br>Probe: Fam-AAATACGAAACCATAGCCTTCCAGCGGC-Tamra
Cytochrome oxidase	For: CGTCGCATTCCAGATTATCCA<br>Rev: CAACTACGGATATATAAGRRCCRRAACTG<br>Probe: Fam-AGGGCATTCCATCCAGCG-Tamra

Supplementary Table 3: List of specific primers used for virus genome resequencing.

Virus	Nucleotide sequence
Penicillium aurantiogriseum totivirus 1	1_For: TTTAAACCCAACCGACACCG<br>1820_Rev: GGGCAACAGAGGCCGTAT
Penicillium aurantiogriseum totivirus 1	1750_For: CTCAGCCCTGGCTGCTAAC<br>3589_Rev: GTCGTCGCTGACTGACTCGA
Penicillium aurantiogriseum totivirus 1	3560_For: CGTGCTTGGCTCGAGTCA<br>4340_Rev: AGTGTTCTGCATGAAGCTAACATAGAG
Penicillium aurantiogriseum totivirus 1	4300_For: TATAATAGCGGGTAATTGGGCTAAC<br>5160_Rev: CCCAAGAATAAGGCCTACCAATC
Penicillium aurantiogriseum partitivirus 1 RNA1	1_For: CTCGAGTCTTTTACCGTGTCAACG<br>1762_Rev: GGATTTTGTTAATATTCTCAGATAAAC
Penicillium aurantiogriseum partitivirus 1 RNA2	1_For: TCCGGGGGGCATGC<br>1576_Rev: CAGGTAAGTAACCCACCCTTTTGTT
